# Human anelloviruses: diverse, omnipresent and commensal members of the virome

**DOI:** 10.1093/femsre/fuaa007

**Published:** 2020-03-19

**Authors:** Joanna Kaczorowska, Lia van der Hoek

**Affiliations:** Laboratory of Experimental Virology, Department of Medical Microbiology, Amsterdam UMC, Location AMC, University of Amsterdam, Meibergdreef 9, Amsterdam, the Netherlands; Laboratory of Experimental Virology, Department of Medical Microbiology, Amsterdam UMC, Location AMC, University of Amsterdam, Meibergdreef 9, Amsterdam, the Netherlands

**Keywords:** Anellome, *Anelloviridae*, anellovirus, commensal virus, orphan virus, torque teno virus

## Abstract

Anelloviruses are small, single stranded circular DNA viruses. They are extremely diverse and have not been associated with any disease so far. Strikingly, these small entities infect most probably the complete human population, and there are no convincing examples demonstrating viral clearance from infected individuals. The main transmission could be via fecal-oral or airway route, as infections occur at an early age. However, due to the lack of an appropriate culture system, the virus–host interactions remain enigmatic. Anelloviruses are obviously mysterious viruses, and their impact on human life is not yet known, but, with no evidence of a disease association, a potential beneficial effect on human health should also be investigated.

## Teaser

Human anelloviruses are understudied viruses that infect the vast majority of the human population; this review visualizes their diversity, discusses the possibility of an ancient origin of anelloviruses and commensal interaction between human anelloviruses and the host.

## INTRODUCTION

Advances in metagenomics have recently presented many novel insights into the microbial world, especially among viruses, with novel species being regularly discovered. Many of these newly discovered viral agents have not been related to any particular disease. In the world of viral science, such viruses are called ‘orphans’, as they lack the ‘parent’—in this case a disease association. A substantial number of human viral ‘orphans’ were found within the family *Anelloviridae*, so-called anelloviruses (AVs). Torque teno virus (TTV) was the first discovered and most studied AV infecting humans, and is currently considered a member of *Alphatorquevirus* genus (Nishizawa *et al*. 1997). The family *Anelloviridae* also includes: torque teno midi viruses (TTMDVs; *Gammatorquevirus*) and torque teno mini viruses (TTMVs; *Betatorquevirus*), which carry a slightly smaller genome than TTVs. The most recent nomenclature includes eleven more genera comprised of only animal isolates. Infections by the *Anelloviridae* family are most probably asymptomatic, with the exception of a member of genus *Gyrovirus*, chicken anemia virus (CAV), which can cause illness in young chickens (Li *et al*. 2017).

Over the years, many different human AVs have been detected in a variety of clinical and environmental samples. It quickly became clear that these viruses are the most abundant eukaryotic viruses in the human virome (Virgin, Wherry and Ahmed 2009). However, the lack of efficient tools considerably tempered the fruitful analysis of virus–host interactions. Currently, there is no cell culture system established to propagate human AVs, and there is no animal model that could provide information on the virus–host interactions. Obtaining an optimal animal model for studying human AVs is problematic. Even though AVs have been identified in a variety of animals, including rodents, no *Mus musculus* (house mouse) AV isolates were reported to date (Nishiyama *et al*. 2014), therefore no mouse model can currently be used to study the biology of AVs.

The virome-related literature has grown *pari passu* with the expansion of metagenomics studies and provided sources of data that may shed more light onto the mystery of human AVs. In this review we summarize the current knowledge about human AVs.

## INFECTION CYCLE OF AVS: GENOME REPLICATION AND PROTEIN SYNTHESIS

### Genome structure and replication

**Figure 1.**
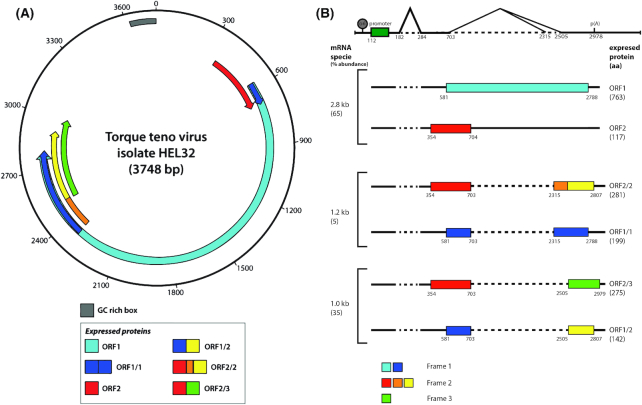
Transcription profile of HEL32 TTV (Genbank accession number AY666122) and the encoded proteins. (**A**) Organization of TTV HEL32 genome. The genome consist of overlapping ORFs in the coding region and a GC-rich box within the noncoding region. Due to the presence of alternative pre-mRNA splicing, 6 different viral proteins are expressed. The ORFs and proteins are indicated with colored boxes. (**B**) The transcript map of TTV HEL32. The polyadenylation signal is indicated with p(A). The percentage relative abundance of each species is indicated, as well as the protein product names and sizes (Qiu, Kakkola *et al*. 2005). The introns are indicated with a dashed line. The figure was adapted from Qiu, Kakkola *et al*. 2005.

The genome of AVs is a single-stranded circular DNA (ssDNA) of negative sense, ranging from 2.0 to 3.9 kb (Miyata *et al*. 1999; Jones *et al*. 2005; Ninomiya *et al*. 2007). The genome contains overlapping open reading frames (ORFs) and an untranslated region (Peng *et al*. 2002) (Fig. 1). There are a few studies which show electron microscopy images of structures presumably representing TTV particles in an infected cell, or TTV particles in immune complexes within an infected individual (Itoh *et al*. 2000; Leppik *et al*. 2007). Based on these micrographs, TTVs are considered to produce non-enveloped icosahedral particles of about 30 nm diameter (Itoh *et al*. 2000).

The majority of known circular DNA viruses, of both prokaryotic and eukaryotic origin, use a so-called rolling circle mechanism to replicate their DNA (Rosario, Duffy and Breitbart 2012), and there is some evidence that AVs use the same mechanism. First of all, the untranslated region of AV contains sequences that may form hairpins to facilitate rolling circle replication (Peng *et al*. 2002; de Villiers *et al*. 2011). TTV genomes encode conserved motifs resembling motifs of rolling-circle replication-associated proteins (Rep proteins) belonging to the *Circoviridae* family (Bendinelli *et al*. 2001). Furthermore, TTV genomes were found in human bone marrow and liver as double-stranded circular DNA, a typical replicative intermediate of rolling circle replication (Okamoto *et al*. 2000b; Okamoto *et al*. 2000d; Wawrzyniak, Plucienniczak and Bartosik 2017). AVs do not encode their own DNA polymerase, thus replication of the genome fully depends on the host cell machinery (Kakkola *et al*. 2007). Therefore, genome replication and conversion of ssDNA into dsDNA occurs in the nucleus.

### Transcription and translation

The transcriptional profile of AVs has been studied by transfection of TTV DNA into cell cultures, but also in bone marrow cells derived from a TTV positive person (Kamahora, Hino and Miyata 2000; Okamoto *et al*. 2000a; Qiu *et al*. 2005; Kakkola *et al*. 2009). Most of the studies were performed using TTV isolate HEL32 (accession number AY666122), which is presently classified as a member of the TTV 3 species. It is important to mention that the transcriptional profiles of other TTVs and especially TTMVs and TTMDVs may differ from the transcriptional profile of TTV-HEL32 that we describe in detail below.

Three species of mRNAs are produced during TTV-HEL32 infection. The largest mRNA can reach 2.8 up to 3.0 kb, while the smaller ones are 1.2 kb and 1.0 kb in length (Fig. 1B). Splicing occurs on one long pre-mRNA, as the mature mRNAs all share the same 5’- and 3’- ends. An intron of approximately 100 nt, localized approximately 70 nt downstream of the 5’end of the mRNA, is not present in all three mRNAs (Qiu *et al*. 2005) (Fig. 1B). The two smaller mRNAs show removal of a second intron, of varying size, due to alternative splicing acceptors.

The longest mRNA is used to translate both the ORF1 protein and the ORF2 protein by initiating at two different AUG codons (Qiu *et al*. 2005). Translation from two initiating AUG codons also occurs for the smaller mRNAs, therefore translation can generate 6 proteins. The 1.2 kb mRNA, which is the least abundant of the mRNAs, is used to translate the ORF2/2 protein and the ORF1/1 protein, while the 1.0 kb mRNA can generate the ORF2/3 and ORF1/2 protein (Fig. 1B) (Qiu *et al*. 2005).

### Protein functions

The ORF1 protein is the largest protein encoded by TTV. It was shown to be localized in the cytoplasm of a cell transfected with a molecular clone of a TTV strain, which indicates a structural role for this protein (Qiu *et al*. 2005). Within the N-terminal, the ORF1 protein contains a stretch of arginine repeats (Erker *et al*. 1999). This arginine rich region is very similar to the ARM motif found in Cap proteins of circoviruses, which is known to possess a DNA binding ability (Erker *et al*. 1999; Sarker *et al*. 2016). This feature might indicate that ORF1 protein, similar to Cap proteins of circoviruses, plays a crucial role in ssDNA packaging (Sarker *et al*. 2016).

The ORF2 protein is most likely a regulatory protein which helps the virus to disrupt immune responses of the host (Zheng *et al*. 2007; Kakkola *et al*. 2009). Interestingly, like the ORF1 protein, this protein is localized exclusively in the cytoplasm, which suggests it is also a structural component (Qiu *et al*. 2005). The ORF2/2 and ORF 2/3 proteins are localized in the nucleus, while ORF1/1 and ORF1/2 proteins are equally present in the cytoplasm and nucleus of transfected cells (Qiu *et al*. 2005). A phosphorylated ORF2/2 protein presumably possesses a DNA template binding capacity, because it contains characteristic serine-rich domains at the C-terminus (Asabe *et al*. 2001). This feature suggests a role of this protein in regulation of genome replication and gene expression (Kakkola *et al*. 2009).

### Ubiquitous viruses

Definitely the most remarkable feature of human AVs is their omnipresence. The first discovered members of the human AV family were TTV isolates detected using molecular biology techniques in the year 1997, in the blood of a Japanese patient showing symptoms of hepatitis of unknown etiology (Nishizawa *et al*. 1997). Since then, AVs have been detected in individuals living on all continents (Spandole *et al*. 2015). It has been hypothesized that probably the entire human population is AV infected, in many cases representing a co-infection with multiple different AV genotypes (Niel, Saback and Lampe 2000; Virgin, Wherry and Ahmed 2009).

AVs have been detected in many biological samples: whole blood, nasal secretions, saliva, bile, feces, tears, semen, breastmilk and urine (Deng *et al*. 2000; Goto *et al*. 2000; Inami *et al*. 2000; Itoh *et al*. 2000; Matsubara *et al*. 2000; Okamoto *et al*. 2000c; Okamoto *et al*. 2000d; Osiowy and Sauder 2000; Schröter *et al*. 2000; Naganuma *et al*. 2008; Kapusinszky, Minor and Delwart 2012; Furuta *et al*. 2015). The presence of AVs in all these materials may cautiously suggest that these viruses exhibit no strong tropism towards a particular cell type or kind of tissue.

Children experience their primary AV infection in the first months of life (Lim *et al*. 2015; Reyes *et al*. 2015; Tyschik *et al*. 2018). It is still not clear whether the first infection is symptomatic or not, however, it was shown that TTV and TTMDV genomes are more prevalent in plasma (*P* = 0.034) and nasopharyngeal swabs (*P* = 0.002) of febrile children compared to afebrile controls (McElvania TeKippe *et al*. 2012). AVs are also frequently detected in older children and adults of all ages (Vasilyev *et al*. 2009; Brassard *et al*. 2015). It is unknown whether the founder virus (or viruses) is maintained in the body, with temporal reactivations (influenced by immune competence, see next paragraph), or whether there is iterative clearance of AVs from the body, with reinfections throughout life (Maggi *et al*. 2001). It is important to mention that it is very difficult to prove the persistence of an infection or the clearance/re-infection hypothesis. A negative result of an AV detection test may indicate absence of the virus but it may also represent a period in which the viral load is below the detection level. One report showed the same type of TTV in samples collected 16 years apart, supporting the theory that people may remain chronically infected with the same TTV variant (Bedarida *et al*. 2017).

Human AVs are most likely repressed by host immunity. It has been stated that AV levels increase together with levels of host immunosuppression. The majority of studies describing this correlation are based on observations in people receiving a solid organ transplant (Burra *et al*. 2008; reviewed in detail by Focosi *et al*. 2016). Intriguingly, Blatter *et al*. found that low AV genome copies are actually associated with transplant rejection or death of pediatric lung recipients (Blatter *et al*. 2018). This may be a direct or an indirect phenomenon, explained by sufficient or insufficient immune suppression, as human AV DNA concentrations increase with the dosage of immunosuppression drugs (De Vlaminck *et al*. 2013; Blatter *et al*. 2018). However, it has to be kept in mind that the increase may actually come from AVs introduced by the transplanted solid organ. Therefore, it is important to look at other conditions that may have an influence on host immunity, for example HIV-1 infection. Unfortunately, the findings in the various studies in HIV-1 infected persons are not consistent. In one study it was found that HIV-1 positive persons display increased TTV and TTMV levels, and the levels were the highest in people that developed AIDS (Thom and Petrik 2007), yet in a larger study this relationship was not found (Nasser *et al*. 2009). A study by Shibayama *et al*. showed TTV levels that were inversely correlated with the levels of CD4 + T lymphocytes in HIV-1-positive persons (Shibayama *et al*. 2001), yet also here this result could not be confirmed, as Moen *et al*. found that CD4 + counts did not significantly correspond with the fluctuations of TTV and TTMV concentrations ((Moen, Sleboda and Grinde 2002)). A study that looked at the excesses during HIV-1 infection: extremely low CD4-counts (<20 CD4 + cells per µl) and high CD4-counts (> 700 CD4 + cells per µl) presented a significant rise in AVs in low CD4 + cell count HIV-1-infected subjects from United States, however, this AV expansion did not reach statistical significance in Ugandan HIV-1 infected subjects with low CD4 + cell counts (Li *et al*. 2013).

The mechanism by which immunity reacts to human AV infection is unknown. A few studies showed that TTV virions in the bloodstream are recognized by immunoglobulins in the blood to form antibody–virus complexes, suggesting that the viruses elicit a humoral immune response (Itoh *et al*. 2000; Tsuda *et al*. 2001; Mankotia and Irshad 2014). The most logical target for immune recognition is the ORF1 protein, that is probably a part of the capsid structure of the virus. Kakkola *et al*. have shown that indeed the ORF1 protein, but also the ORF2 protein, are recognized by antibodies present in the serum of TTV-positive persons (Kakkola *et al*. 2008).

In case human AVs persist in a host, it is likely that they have developed mechanisms that allow them to remain present. The ORF2 viral protein may be involved, as the protein has the capability to inhibit the NFκB pathway (Zheng *et al*. 2007). Additionally, TTV encoded microRNAs may target the host mRNAs encoding N-myc and STAT interactor, responsible for modulating the interferon and cytokine signaling pathways (Kincaid *et al*. 2013). Moreover, it was recently found that TTV particles can circulate in the body within exosomes (Martelli *et al*. 2018). Travelling within vesicles can promote spread of the virus by two means: it may enhance infectivity by allowing entry of TTVs in otherwise non-permissive cells, and the virus inside the exosomes may be less exposed to neutralizing antibodies (Martelli *et al*. 2018).

### Diversity of human anelloviruses

**Figure 2.**
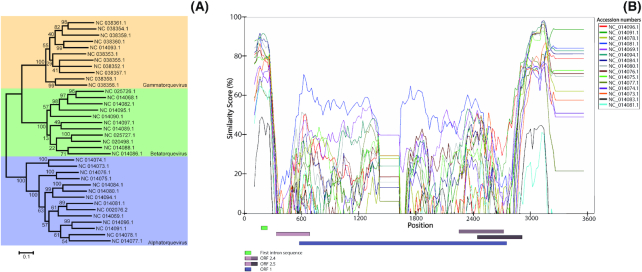
Analysis of genetic variability of *Anelloviridae*. (**A**) Maximum-likelihood phylogenetic tree of full-length nucleic acid sequences of reference isolates of TTV, TTMV and TTMDV. (**B**) Similarity plot of full-length reference nucleotide sequences of TTV. TTV 1 sequence (accession: NC 0 02076.2) was used as a query. Similarity plots of nucleotide sequences of TTMV and TTMDV are shown in supplementary Figure S1. The list of accession numbers of nucleotide sequences used in the analysis is shown in supplementary Table S1. The similarity score of all plots was calculated using Kimura model. The predicted ORFs of each query are indicated with colored boxes below the plot. The similarity plots were constructed using SimPlot software (Lole *et al*. 1999).

**Figure 3.**
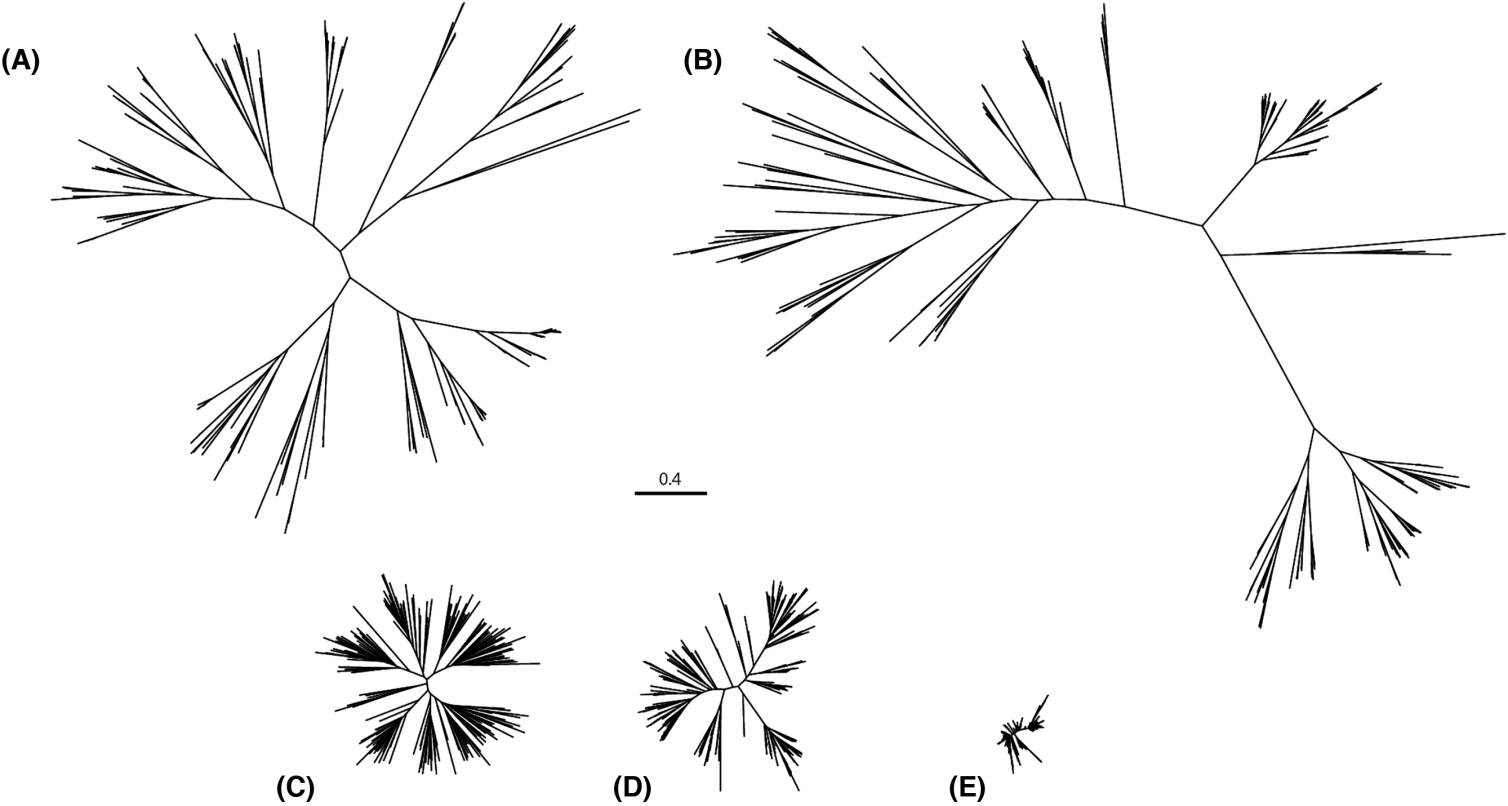
Phylogenetic analysis of amino acid sequences of structural proteins of human TTV, HPV, HIV-1 group M, HCV and HBV. A comparison of evolutionary distances of amino acid sequences of structural proteins of: (**A**) human TTV (ORF1 protein), (**B**) HPV (minor capsid protein L2), (**C**) HIV-1 group M (envelope polyprotein gp160), (**D**) HCV (E2 protein), (**E**) HBV (large surface protein LSP). Only sequences derived from human isolates were selected in this analysis. The trees were generated using a workflow presented in supplementary Figure S2. The list of accession numbers of nucleotide sequences used in the TTV phylogenetic tree construction are listed in supplementary Table S2. Used tools and databases are listed in supplementary Table S3 (Miller *et al*. 2010; Katoh *et al*. 2017).

The TTV genus contains 29 species (ICTV, state for July 2018), and a cut-off value of 35% nucleotide identity within the *ORF1* gene is applied as a species distinction criterion (Maggi and Bendinelli 2009; Biagini *et al*. 2011). The coding region is considered the most variable part of the genome, (Nishizawa *et al*. 1999; Kakkola *et al*. 2008) visualized for TTV genomes in Fig. 2, and for TTMV and TTMDV genomes in supplementary Figure S1. The diversity of AVs is especially striking in comparison with other viral families. In Fig. 3, the genetic diversity of several human viruses is shown: hepatitis C virus (HCV), human immunodeficiency virus 1 (HIV-1) group M, hepatitis B virus (HBV), human papillomavirus (HPV) and human TTV. The human TTVs show a striking genetic diversity (Fig. 3A), comparable to the diversity seen for HPV (Fig. 3B). In order to compare these two groups, the mean distances between the aligned amino acid sequences were calculated in MEGA v.6.06 using a Poisson correction model (Tamura *et al*. 2013). The mean distance of the TTV phylogenetic tree in Fig. 3 is 0.633 amino acid substitutions per site, while for HPV it reaches a value of 0.827 amino acid substitutions per site (Table 1). Both values are very high compared to those observed for HIV-1 group M, HCV and, especially, HBV (Table 1). HPVs are estimated to have diverged from their common ancestor approximately 75 million years ago, prior to the appearance of *Homo sapiens* (Van Doorslaer 2013). The same may be true for the AVs, as human and non-human primate AV isolates cluster phylogenetically (Fahsbender *et al*. 2017), similar to clustering among human and primate HPVs (Van Doorslaer 2013). This suggests that *Anelloviridae* is an ancient virus family, and millions of years of evolution have led to the current genetic diversity.

**Table 1.** Mean genetic distances of the Fig. 3 phylogenetic trees. The mean distances between the aligned amino acid sequences were calculated in MEGA v.6.06 using a Poisson correction model (Tamura *et al*. 2013).

Virus (protein)	Mean genetic distance (aa substitutions per site)
Human TTV (ORF1 protein)	0.633
HPV (minor capsid protein L2)	0.827
HIV-1 group M (envelope polyprotein gp160)	0.283
HCV (E2 protein)	0.296
HBV (large surface protein LSP)	0.083

An alternative explanation for the high diversity of AVs would be fast evolution due to a high mutation rate, or frequent recombination between virus strains. A high mutation rate seems unlikely as AVs use host polymerases for their replication (Kakkola *et al*. 2007), enzymes that have an efficient proofreading capacity. Recombination is also mentioned as an explanation since full length TTV genomes indeed show signs of recombination (Worobey 2000; Fahsbender *et al*. 2017). Although interesting, the presence of recombination events between strains cannot easily explain the huge variation among human AVs visible today. Hypotheses like genomic rearrangements and/or the influences of repetitive sequences have also been proposed (Leppik *et al*. 2007; Kakkola *et al*. 2008). Yet, there is no proof that these mechanisms have contributed to the genetic diversity of AVs.

The mutation rate of viruses can be estimated by studying viral sequences isolated at different time points from the same host. Longitudinal studies focusing on within host evolution of human AVs are scarce. In one study, three serum samples were collected 12 years apart from an HCV-positive patient with a chronic TTV infection (Umemura *et al*. 2002). The mutation rate was estimated to be approximately 7 × 10^−4^ substitutions per site per year within *ORF1* and *ORF2* genes (Umemura *et al*. 2002). In another study, serial cat and human samples were collected 6.5 and 16 years apart, respectively (Bedarida *et al*. 2017). For both sample sets, mutation rates reaching approximately 2 × 10^−4^ nucleotide substitutions per site per year were calculated. The substitution rates detected in both studies are relatively high for a DNA virus, however, similarly high rates have been calculated for other common ssDNA viruses: parvoviruses (human parvovirus B19 and canine parvovirus 2) and plant-infecting *Geminiviridae* (Duffy *et al*. 2008). The mutation rates of small ssDNA viruses, including AVs, can therefore be placed in between the slow mutating dsDNA viruses and rapidly mutating RNA viruses. However, it is important to keep in mind that in the study of Bedarida *et al*., as well as in the study of Umemura *et al*., no replicates of the clinical samples were tested (Umemura *et al*. 2002; Bedarida *et al*. 2017). This is important in order to rule out generation of hybrid sequences that may occur during PCR. Well-controlled studies with larger sample sets and more time points are essential for future studies to reveal whether AVs are fast or slow evolving viruses.

### Potential infection routes

As already mentioned, AVs are found in a large range of biological specimens, like blood, semen, nasal secretions, saliva and feces (Goto *et al*. 2000; Inami *et al*. 2000; Itoh *et al*. 2000; Osiowy and Sauder 2000; Kapusinszky, Minor and Delwart 2012). Therefore, a variety of transmission routes including fecal-oral, airway, sexual contact, blood-blood, solid organ transplantations and vertical transmission are all possible for AVs. Air-mediated transmission by saliva droplets may be a likely transmission route of the first infection, since (1) the levels of TTV in the nasal cavity and in saliva exceed that in blood significantly (Deng *et al*. 2000; Maggi *et al*. 2003), and (2) children get infected at a very early age, excluding the blood transfusion or sexual transmission as major infection routes at this age (Ninomiya *et al*. 2008; Lim *et al*. 2015). One study with longitudinally collected samples was performed on a child who was initially TTV positive in the nasal cavity and negative in the blood compartment (Maggi *et al*. 2003). One month later, the child became positive in blood, and the genotype of the virus isolated from both compartments was identical. Airway epithelium may thus be a primary site of TTV entry.

It is still not clear whether AVs can pass the placenta and infect fetuses. Low loads of TTV DNA were detected in umbilical cord blood and peripheral blood of infants born to TTV infected mothers (Gerner *et al*. 2000; Matsubara *et al*. 2001), however, contaminations with minute quantities of blood from the mother during cord blood withdrawal could not be excluded. A more recent study using cord blood from 84 children born to mothers carrying TTVs did not find TTV DNA in cord blood (Tyschik *et al*. 2017). It is therefore regarded less likely that AV transmission occurs via the placenta. The transmission may however occur at the moment of delivery, when the newborn has direct contact with the blood and vaginal fluids of the mother. The levels of TTVs are generally higher in infants born naturally compared to ones delivered via caesarian section (McCann *et al*. 2018). Transmission via breast milk is another route the virus may use to infect a child. However, a study by McCann *et al*. showed that AV levels in children are not influenced by the breastfeeding status (McCann *et al*. 2018). In accordance with this, another group reported that TTV-presence in mother's blood or milk is not a risk factor for infection of the infant (Ohto *et al*. 2002).

### Anelloviruses in culture

Since AVs were discovered, many groups have tried to set up an efficient cell culture system to amplify and investigate these viruses and their interaction with host cells. So far there is not a robust culture system where, following infection or transfection, sufficient free progeny virus is produced to support replication upon passaging of the cell-free virus. Some studies that isolated peripheral blood mononuclear cells carrying TTVs directly from hosts showed an increase in the concentration of intracellular TTV DNA after stimulation of the cells (Okamoto *et al*. 2000c; Mariscal *et al*. 2002). In another study Chang liver cells were infected with TTVs from a pool of serum samples (Desai *et al*. 2005). Viral transcripts were detected in the cells, and the virus DNA concentration increased, yet passaging of the virus only succeeded as a co-culture of the infected Chang cells, and not as a cell-free progeny virus. The only two systems capable of passaging cell-free virus was with peripheral blood mononuclear cells and a leukocyte cell line (Raji cells), yet for both systems only a single passage of the virus was presented (Desai *et al*. 2005).

A few research groups attempted transfection of cell lines with TTV molecular clones. DNA transfection is a commonly used method to enhance virus replication. It was successfully used for ‘unculturable’ viruses such as HPVs (Meyers, Mayer and Ozbun 1997), and also for veterinary relevant pathogenic circular viruses, such as chicken anemia virus (CAV) and porcine circovirus 2 (PCV-2) (Noteborn *et al*. 1991, Meehan *et al*. 1998). In one of the first transfection studies, 293T cells showed the highest rate of human TTV DNA replication. Virus culture supernatants could be passaged, yet the amount of replicative viral DNA decreased considerably after passaging (Kakkola *et al*. 2007). Lymphoma and T-cell leukemia cell lines (L428) were also successfully transfected with full-genome molecular clones of two TTV isolates (Leppik *et al*. 2007). However, long-term replication of TTVs was not achieved (Leppik *et al*. 2007). In a follow-up study a 293TT cell line that expresses the simian virus 40 (SV40) large-T antigen was used (de Villiers *et al*. 2011). That study reported some success following transfection and subsequent passaging of the virus. Noticeably, the passage was effective only when infected cells were used in co-culture with new target cells, and not with cell-free supernatant containing released viral particles. Moreover, the levels of TTV DNA decreased with each subsequent passage (de Villiers *et al*. 2011). Another study attempted cultivating human TTMV both in 293T and the alveolar epithelial cell line A549 (Galmes *et al*. 2013). These cell lines were transfected with molecular clones of three TTMV species and virus replication was observed in both cell lines. The A549 cells could even be infected with post-transfection supernatants from both 293T and A549 cells (Galmes *et al*. 2013). Unfortunately, there is no information on further attempts to passage the virus.

It has been suggested that Epstein-Barr virus (EBV) might have a helper function for TTV infection (Borkosky *et al*. 2012). Many variants of hematopoietic EBV-positive and EBV-negative cell lines were therefore tested in transfection experiments, along with Burkitt's lymphoma cell lines and B cell lines (Borkosky *et al*. 2012). Replication was observed and the level of TTV replication was substantially higher when EBV was present in the cells (Borkosky *et al*. 2012), yet there was no mention of a successful passage of progeny virus.

### Sneaky foes or good friends?

Since their discovery, scientists have tried to link AV infection to a disease. Hepatitis, respiratory disease, autoimmune disorders and even certain cancers have been mentioned. Yet, the fact that children experience their first infection at an early age (Lim *et al*. 2015; Reyes *et al*. 2015; Tyschik *et al*. 2018), and that probably most humans are either chronically infected or continuously re-infected, reduces the likelihood that AVs are truly causing disease. One may even consider looking from a completely different perspective. As in the case of a balanced gut microbiome, which is needed for a healthy intestinal microenvironment, it may be that the AV population is part of a personal virus flora that is positively influencing human physiology. The idea of a ‘beneficial virome’ is not completely new. Murine norovirus is an example of a vertebrate virus that has a positive influence on its host by shaping the immunity and sustaining the homeostasis. The infection with this virus inverts the destructive influence of antibiotic treatment on germ-free mouse gut and protects from superinfection with bacterial pathogens (Kernbauer, Ding and Cadwell 2014). Bodily functions that would benefit from AV infection are currently unknown, but it can be hypothesized that it has to do with to shaping of the immunity in the first year(s) of life.

The richness of AV species increases in healthy infants gut until approximately 12 months of age, and after the 15th month of life it starts to decrease (Lim *et al*. 2015; Reyes *et al*. 2015). In addition to that, the levels of TTV DNA raise in blood of newborns starting in the second month of life (Tyschik *et al*. 2018). The high level of AVs in this period certainly has an influence on the development of immune system and its maturation, since fluctuations in immune cell and cytokine levels are of high importance for shaping immunity (Round and Mazmanian 2009; Virgin, Wherry and Ahmed 2009). AVs have been detected in cervical samples from healthy women (Fornai *et al*. 2001) and from healthy pregnant women (Chan *et al*. 2001). An intriguing link was found between lowered AV levels during pregnancy and a risk of a child developing schizophrenia (Canuti *et al*. 2015). It was hypothesized that the lowered AV can be a sign of an activated immunity of the mother, possibly related to psychotic disorders developing in the offspring. We therefore favor the idea that AV infection in the first months of human life is an important, natural event.

### Concluding remarks—counting the missing pieces of the puzzle

It has been more than twenty years since the discovery of the first member of the family *Anelloviridae*, yet two decades of research on this intriguing group have resulted in more questions than answers. One of the main mysteries remaining to be solved is the lack of clinical symptoms observed upon AV infection. Despite studies describing associations with diseases, ranging from hepatitis to cancer, no study has yet provided the essential proof of involvement in disease. A robust virus culture system, or an animal model, is currently not available. In this review we theorize that a link with a specific disease is unlikely. In addition to the lack of disease association, the fact that probably all humans are carrying AVs from an early age fortifies the idea that AV infections are not causing disease. A long history in virus-host interaction with subsequent adaptation can explain the absence of symptoms upon AV infection, as well as the large genetic variation among strains.

In the gut, a microbiome can be associated with health, and one might propose that AVs in the body are the systemic viral counterpart. Each person may carry an ‘anellome’, which could be either stable or variable in time. If the anellome is stable, this may be an indication that it is a personalized and healthy part of the virome, similar to the microflora of the human gut where a balanced and diverse microbiota is associated with health. In this scenario it is envisioned that new AV entries that occur throughout life are not replacing the variants that persons carry from birth. An alternative hypothesis is that re-infections with new AV types and clearance happen frequently and that the immune system is in a constant battle with a changing anellome. The current opportunities that metagenomic techniques provide, combined with available biobanks that stored clinical material since the 1980s as part of surveillance programs, will probably shed light on this dilemma in the foreseeable future.

## Supplementary Material

fuaa007_Supplemental_Files
