# Supplementary material for: Human anelloviruses: diverse, omnipresent and commensal members of the virome
Source: FEMS Microbiol Rev. 2020 Mar 19;44(3):305–13. doi: 10.1093/femsre/fuaa007 (PMC7326371; doi:10.1093/femsre/fuaa007)
Supplement: fuaa007_Supplemental_Files [file fuaa007_supplemental_files.zip › Supplement_clean.docx]

# Supplementary data

Figure S1: Analysis of genetic variability of two anellovirus genera: TTMV (*Betatorquevirus*) and TTMDV (*Gammatorquevirus*). A) Similarity plot of full-length reference nucleotide sequences of TTMV. TTMV 1 sequence (accession: NC 014097.1) was used as a query. B) Similarity plot of full-length reference nucleotide sequences of TTMDV. TTMDV 2 sequence (accession: NC 01093.1) was used as a query. The list of accession numbers of nucleotide sequences used in the analysis is shown in supplementary Table S1. The similarity score of all plots was calculated using Kimura model. The predicted ORFs of each query are indicated with colored boxes below the plot. The similarity plots were constructed using SimPlot software.

Figure S2: The workflow of the phylogenetic analysis comparing the genetic distances of TTVs and four other viral groups. The full list of used software and viral databases are presented in the supplementary Table S3.

Table S1: The list of names and accession numbers of sequences that were used in the full-length phylogeny and SimPlot analysis (Figure 2). All the mentioned sequences are the reference sequences available in Genbank (state for December 2019).

| Sequence name | Accession number |
| --- | --- |
| Torque teno midi virus 15 DNA, complete genome, isolate: Pt-TTMDV210 | NC_038362.1 |
| Torque teno midi virus 14 DNA, complete genome, isolate: MDJN97 | NC_038361.1 |
| Torque teno midi virus 7 DNA, complete genome, isolate: MDJHem3-2 | NC_038354.1 |
| Torque teno midi virus 13 DNA, complete genome, isolate: MDJN69 | NC_038360.1 |
| Torque teno midi virus 2, complete genome | NC_014093.1 |
| Torque teno midi virus 6 DNA, complete genome, isolate: MDJHem3-1 | NC_038353.1 |
| Torque teno midi virus 8 DNA, complete genome, isolate: MDJN1 | NC_038355.1 |
| Torque teno midi virus 5 DNA, complete genome, isolate: MDJHem2 | NC_038352.1 |
| Torque teno midi virus 12 DNA, complete genome, isolate: MDJN51 | NC_038359.1 |
| Torque teno midi virus 10 DNA, complete genome, isolate: MDJN14 | NC_038357.1 |
| Torque teno midi virus 11 DNA, complete genome, isolate: MDJN47 | NC_038358.1 |
| Torque teno midi virus 9 DNA, complete genome, isolate: MDJN2 | NC_038356.1 |
| Torque teno mini virus ALH8, complete genome | NC_025727.1 |
| TTV-like mini virus isolate TTMV_LY1, complete genome | NC_020498.1 |
| Torque teno mini virus 3, complete genome | NC_014088.1 |
| Torque teno mini virus 2, complete genome | NC_014086.1 |
| Torque teno mini virus ALA22, complete genome | NC_025726.1 |
| Torque teno mini virus 8, complete genome | NC_014068.1 |
| Torque teno mini virus 7, complete genome | NC_014082.1 |
| Torque teno mini virus 6, complete genome | NC_014095.1 |
| Torque teno mini virus 1, complete genome | NC_014097.1 |
| Torque teno mini virus 5, complete genome | NC_014089.1 |
| Torque teno mini virus 4, complete genome | NC_014090.1 |
| Torque teno virus 15, complete genome | NC_014096.1 |
| Torque teno virus 16, complete genome | NC_014091.1 |
| Torque teno virus 19, complete genome | NC_014078.1 |
| Torque teno virus 3, complete genome | NC_014081.1 |
| Torque teno virus 1, complete genome | NC_002076.2 |
| Torque teno virus 4, complete genome | NC_014069.1 |
| Torque teno virus 6, complete genome | NC_014094.1 |
| Torque teno virus 8, complete genome | NC_014084.1 |
| Torque teno virus 7, complete genome | NC_014080.1 |
| Torque teno virus 10, complete genome | NC_014076.1 |
| Torque teno virus 12, complete genome | NC_014075.1 |
| Torque teno virus 14, complete genome | NC_014077.1 |
| Torque teno virus 27, complete genome | NC_014074.1 |
| Torque teno virus 28, complete genome | NC_014073.1 |

Table S2: List of TTV species selected for the phylogenetic analysis shown in Fig. 3 A. The sequences were selected from the list of species presented on the ICTV website (state for December 2019).

| Sequence name | Accession number |
| --- | --- |
| Torque teno virus 1-TA278 | AB008394 |
| Torque teno virus 3-HEL32 | AY666122 |
| Torque teno virus 5-TCHN-C1 | AF345523 |
| Torque teno virus 6-KAV | AF435014 |
| Torque teno virus 7-PMV | AF261761 |
| Torque teno virus 8-Kt-08F | AB054647 |
| Torque teno virus 9-BM1C-18 | DQ187006 |
| Torque teno virus 10-JT34F | AB064607 |
| Torque teno virus 11-TCHN-D1 | AF345524 |
| Torque teno virus 12-CT44F | AB064605 |
| Torque teno virus 13-TCHN-A | AF345526 |
| Torque teno virus 15-TJN01 | AB028668 |
| Torque teno virus 16-TUS01 | AB017613 |
| Torque teno virus 17-SENV-G | AX025830 |
| Torque teno virus 18-SENV-C | AX025718 |
| Torque teno virus 19-SANBAN | AB025946 |
| Torque teno virus 20-SAa-10 | AB060594 |
| Torque teno virus 21-TCHN-B | AF348409 |
| Torque teno virus 22-svi-1 | AX174942 |
| Torque teno virus 23-CH65-2 | AB049607 |
| Torque teno virus 24-SAa-01 | AB060597 |
| Torque teno virus 27-CT23F | AB064595 |
| Torque teno virus 28-CT43F | AB064598 |
| Torque teno virus 29-yonKC009 | AB038621 |

Table S3: The list of software and databases used in the phylogenetic and similarity plot analysis.

| Tool or database | Reference and link |
| --- | --- |
| International Committee on Taxonomy of Viruses (ICTV) (last updated in July 2018) | https://talk.ictvonline.org/ |
| Protein-protein BLAST (Blastp) | https://blast.ncbi.nlm.nih.gov/Blast.cgi?PAGE=Proteins |
| CodonCode Aligner version 6.0.2 | https://www.codoncode.com/index.htm |
| MAFFT version 7 | (Katoh, Rozewicki et al. 2017)  https://mafft.cbrc.jp/alignment/server/ |
| CIPRES version 3.3 | (Miller, Pfeiffer et al. 2010)  http://www.phylo.org/ |
| FigTree version 1.4.3 | http://tree.bio.ed.ac.uk/software/figtree/ |
| HPV databases | https://pave.niaid.nih.gov/ |
| HIV databases | https://www.hiv.lanl.gov/content/index |
| HCV databases | https://hcv.lanl.gov/content/index |
| HBV databases | https://hbvdb.ibcp.fr/ |
| MEGA version 6.06 | (Tamura, Stecher et al. 2013) |
| SimPlot version 3.5.1 | https://sray.med.som.jhmi.edu/SCRoftware/simplot/  (Lole *et al*., 1999) |
