# Supplementary figures and images for: Human anelloviruses: diverse, omnipresent and commensal members of the virome
Source: FEMS Microbiol Rev. 2020 Mar 19;44(3):305–13. doi: 10.1093/femsre/fuaa007 (PMC7326371; doi:10.1093/femsre/fuaa007)

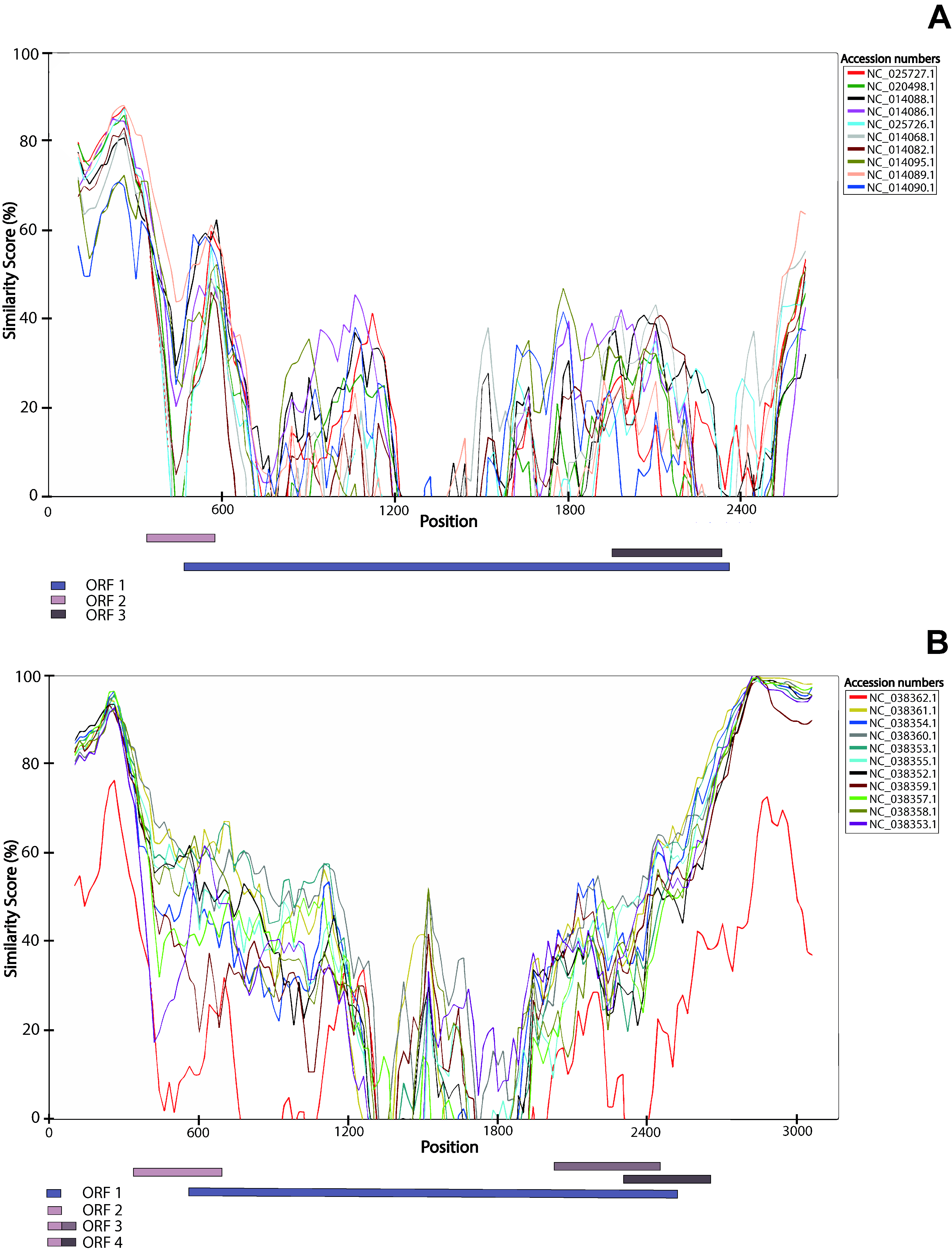

Supplement: fuaa007_Supplemental_Files [file fuaa007_supplemental_files.zip › Figure_S1.tif]

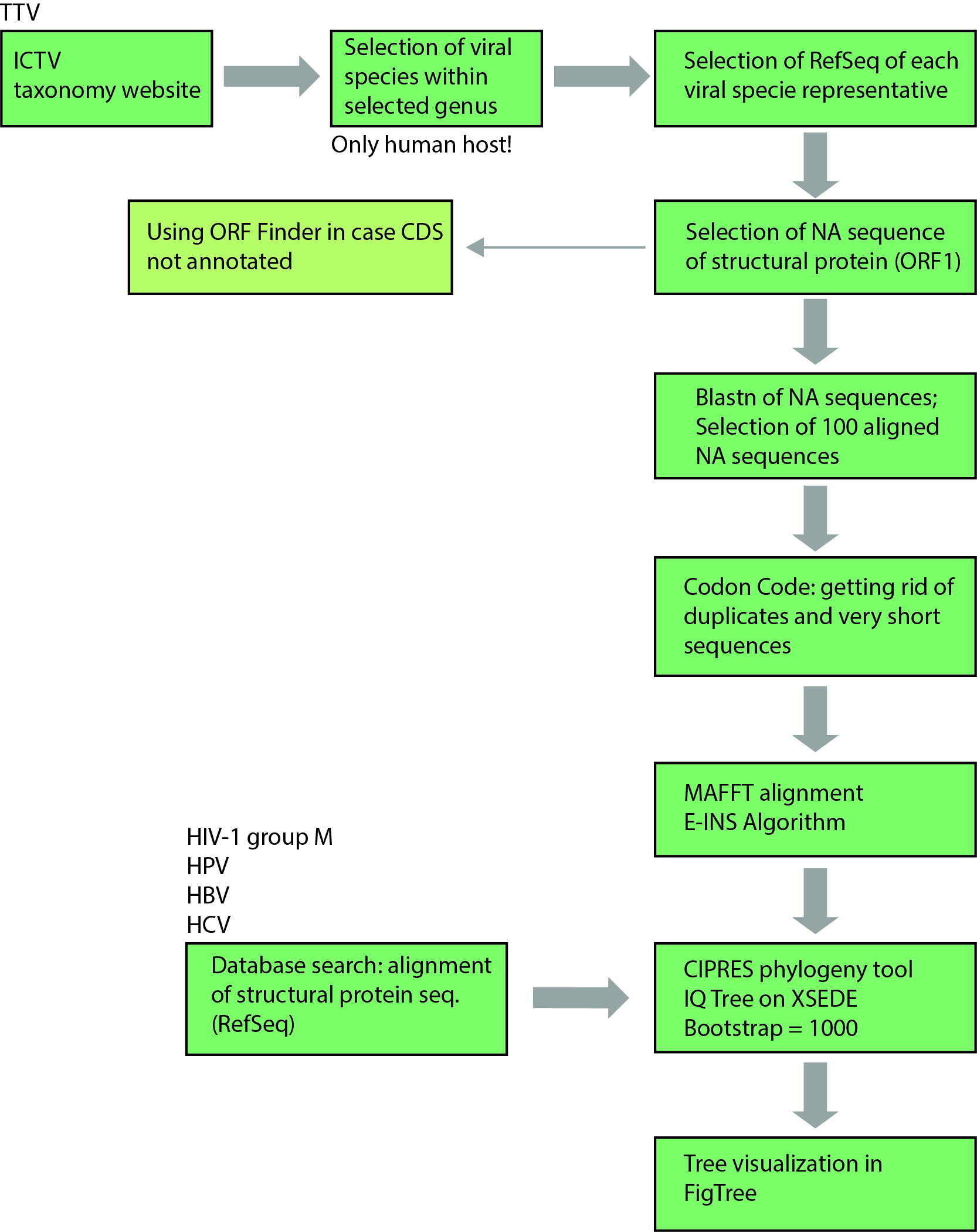

Supplement: fuaa007_Supplemental_Files [file fuaa007_supplemental_files.zip › FigureS2.tif]
